# Speculation about Options for Teen Tobacco Use Cessation in the Russian Federation

**DOI:** 10.1186/1617-9625-3-3-1

**Published:** 2007-02-15

**Authors:** Steve Sussman, Ulya Gufranova, Andrey Demin

**Affiliations:** 1 University of Southern California, Schenenov Moscow Medical Academy, and Russian Public Health Association

## Abstract

This paper summarizes prevalence and consequences, recent policies, prevention and cessation efforts, recent developmental work (focus groups), and speculation about the current status of cigarette smoking in the Russian Federation. Unique aspects of modern Russian society are suggested as leading to relatively high prevalence internationally of smoking among Russian males. Similar factors may lead to deflated smoking cessation attempt and quit rates. We believe that the future of tobacco control in Russia is close, but that it will involve raising the prices of tobacco products, enforcing no tobacco use policies among minors, ratification of the Framework Convention on Tobacco Control (FCTC) and implementation of evidenced-based tobacco use prevention and cessation programs.

## 

"Today I intend to tell you about tobacco – like a people's whim and disaster."

I. M. Dogel, a Russian pharmacologist.

The negative influence of tobacco on health currently is one of the most thoroughly investigated health problems. It is known today that there is a cause-and-effect relation between smoking and many diseases, particularly cardiovascular, pulmonary, and cancerrelated diseases. The recognition that tobacco use is the leading behavioral cause of death has led, directly or indirectly, to smoking prevalence being cut in half in several countries in the world [10]. Tobacco control efforts are relatively new in the Russian Federation. Russia is the largest country geographically with a very long and proud history. However, currently the average life span is one of the lowest in the world, particularly among men, due in large part to smoking. Russia like other countries deserves a healthy posterity.

"The veritable civilization index isn't a reach level and towns dimensions, not harvest abundance, but a person's look which was cultivated by country" (R.U.Emerson, American poet and philosopher in 19th century). The look cultivated by a country hooked on nicotine is not admirable. Cigarette smoking in Russia has led to relatively high work absences, decreased productivity, decreased longevity, high national medical costs, decreased personal disposable income, and has contributed to a social fatalism that has immobilized economic growth among the masses. People have to understand that their health is open to a type of injury that is within their behavioral control. "The secret of life prolongation is the ability to not shorten it" (Feihttersleben, Australian doctor in the 19th century). As Russian doctor and scientist E.M. Tareev said, "The State must demand from citizens that they don't shorten their life from smoking. The community which leads a rational and hard-edged fight with harmful habits promotes a national recovery."

In Russia, for people over 18 years old, approximately 60% of adult males smoke and 16% of adult females smoke [10,16]. Smoking among adult males in the Russian Federation is among the highest prevalence in the world (after Armenia, Cambodia, China, Kenya, and Turkey), whereas it is much lower among adult females. On the other hand, prevalence of smoking among young adult women may be as high as 27% ([7,16] WHO, 1999), and women in Russia may tend to grossly underestimate their smoking prevalence [9]. Russian families may spend an average of 15% of their total household budget on cigarettes [16].

Over 50% of cigarettes sold in Russia are produced by transnational companies [4,16]. These are located primarily outside of the Russian Federation, including Japan Tobacco International, British American Tobacco, Liggett, Imperial, and Phillip Morris. Domestic companies include Tabakprom and Yelets, the latter which produces the popular local brand, Prima. Perhaps 20% of cigarettes on the market are sold illegally. The cost of a pack of imported and domestic cigarettes is approximate $1 and $0.62 USD, respectively, and cost of a Belomorkanal papirosi (an inexpensive local-type cigarette, 5^th ^grade tobacco smoked through a cardboard tube) is $.07 USD. Thus, tobacco smoking is relatively inexpensive.

In 2000, in the Russian Federation, the proportion of lung cancer deaths attributed to smoking was 94% among males and 40% among females. The proportion of cardiovascular disease due to smoking was 27% among males and 2.3% among females (e.g., [3]). The proportion of total deaths through all related causes due to smoking was 26% among males, 3% among females and 15% of total population deaths. There are 300,000 tobacco-related deaths in the Russian Federation per year. Average number of years lost due to smoking is 19 among males and 16 among females [10]. The World Health Organization states that tobacco and alcohol use are the leading risk factors for premature deaths in the Russian Federation http://www.who.dk/eprise/main/WHO/Progs/CHHRUS/sum/20041126_2.

One means to ascertain a trend of decreased prevalence is through assessments of youth. Unfortunately, in some estimates, one third of Russian youth have tried a cigarette by 10 years of age, particularly males [15,16]. The median age of smoking initiation is 11 years. A total of 27.4% of 15 year old males and 8.5% of 15 year old females are current smokers (and smoke an average of 17 days each month, approximately 6 cigarettes per day when smoking; 9% of 11–15 year old boys and 6% of same age girls are daily smokers; [16]). Up to 27% of high school youth are daily smokers depending on region sample and average age (e.g., The Global Youth Tobacco Survey Collaborative Group, 2002; [8,16]). Only 4% use a tobacco product other than cigarettes. Pessimistically, over half of nonsmoking high school youth report contemplation of smoking in the future and 45.8% of Russian teens are regularly exposed to passive smoking at home [8,10,16]. These data indicate that addiction to nicotine occurs at a young age, that prevalence of smoking among youth is high. Perhaps, information can be learned about the context of teen smoking that would assist in smoking cessation efforts.

## Contexts of Smoking among Youth: Reflection of Policy Enforcement

Regarding locations of smoking, approximately 44% smoke in public spaces (e.g., on the street, in parts, near stores), 14% smoke at social events, 7% smoke while at the high school, and 6% smoke at home [16]. A total of 63% purchase cigarettes in a store or from a street vendor, 7% buy cigarettes from vending machines, 5% receive cigarettes as gifts from older persons, and 17% borrow cigarettes from a peer. Youth pay about $0.60 U.S. (50 rubles) per pack of cigarettes [16]. Thus, high school youth spend approximately $60 U.S.D. per month on cigarettes. If this amount spent is accurate (and another family member smoked; e.g., the parent), 15% of an average family's income is spent on the youth's and other person's cigarettes (based on a sample of 99 schools in Moscow in 1999; [16]). The most popular single brand of cigarettes smoked among youth is Golden Jawa (20%), followed by L&M (14%), and Marlboro (12%). However, most youth do not have a strong brand preference [16].

The data suggest operation of a rather lax enforcement of youth smoking policy. While youth under 18 years of age are prohibited from purchasing tobacco, and store clerks that sell to minors may be subject to an administrative hearing and sanctions according to the current Federal Law on Tobacco (passed in 2001; see Appendix 1), no penalties for selling tobacco to youth are evident. In fact, 63% of Moscow school students purchased tobacco from street vendors in 1999; little decrease is noted since then [4,16]. A total of 17% of high school age youth report having been offered free cigarettes by cigarette company representatives, and 50% are well aware of tobacco advertising [16]. Parents might be prosecuted for knowingly permitting their youths to purchase cigarettes but this policy would be difficult to enforce because proof would be difficult to obtain. Smoking is restricted but not totally banned in several public locations (workplaces, covered sports facilities, public transport, air flights, cultural and educational institutions and government buildings). Size of warning labels on packages is small (about 4% of the pack). Over 80% of Russia's youth are aware of the change in laws and 90% accept the law (see Appendix 1). However, there is a general attitude that the law can't be enforced.

Tobacco industry taxes only accounts for around 2% of the federal budget, which is relatively small [16]. In 2000, the excise tax on cigarettes was only approximately 7% of the retail price [16]; the current total tax on cigarettes may be 32% of the total cost which is among the lowest in the world [10]. There would seem reason considering the toll on productivity to attempt large tax increases and widespread anti-tobacco programming though this has not happened. There are publicized ongoing lawsuits again the tobacco industry in the Russian Federation ([10,16] with causing suffering, hiding risks, hurting the economy themes). The outcomes of these lawsuits wait to be seen.

There are some changes being pursued that might affect youth smoking through reduction in social modeling of smoking or reduction in access to cigarettes. Delegates of the State Duma, Vladimir Medinskiy and Nikolai Gerasimenko, are attempting to implement some changes to the Federal Law about Restriction of Smoking of Tobacco (the original law is shown in Appendix 1). They are pursuing to increase preventive inscriptions on a cigarettes pack up to 50% of the size of the pack, create restricted smoking areas in restaurants, change contents of tobacco products to conform to international standards (in nicotine and tar content), and prohibit smoking on all air flights ("KOMMEPCTaHTъ" (a Russian newspaper; May 29, 2006; Look Business Newspaper web site, October 11, 2006; http://vz.ru).

We have begun a U.S.-Russian program of research on teen smoking cessation as of May, 2006. We focus on this topic next. We address the plausibility of tobacco use cessation programming among youth in the Russian Federation. Currently, there are a few programs for the prevention of tobacco dependence. However, it would appear that very few teen tobacco use cessation programs have been examined or offered in the Russian Federation. Also, there are no quit lines. Nicotine Replacement Therapy (NRY) is available over the counter but there is no government coverage of cost. There are no interventions to protect non-smokers. We might ask what types of programming may impact youth smoking and perhaps break the chain of smoking prior to adulthood.

## Available Literature

### Program Options

The most exhaustive review of Russian teen smoking in the English language was completed by Ross (2004). In her examination of Global Youth Tobacco Survey responses in 1999, she observed that 70% of Moscow high school smokers desire to quit smoking, but only 8% report having received professional help to quit. Her results, along with Demin's (2001), suggest that at least ten strategies should be implemented to decrease prevalence of teen smoking among Russian youth. First, higher prices should be charged for cigarettes (currently smoking is affordable among youth). Indeed, higher cigarette prices appear associated with a lower smoking prevalence across age groups (with a price elasticity of conditional demand approximately -.50) and fewer cigarettes smoked each month. Second, appeals to males not to smoke are needed, since smoking appears to be identified with Russian male values and life struggles. Third, classroom discussion about smoking should be conducted regularly, as any open discussion in classes may be associated with decreased prevalence [16]. Fourth, offers of free cigarettes by cigarette-company representatives should be outlawed and otherwise discouraged. Fifth, related, cigarette sales to minors should be banned and enforced. Sixth, vending machines should be banned. Easy access to cigarettes simply makes it more possible for youth to experiment and become addicted to smoking. Seventh, tobacco advertising should be restricted. For example, sponsorship-related advertising and tobacco billboards should be outlawed. Eighth, counteradvertising should be encouraged. Ninth, no-smoking policies in public buildings should be both enacted and enforced. Finally, both evidence-based prevention and cessation programs should be developed and disseminated.

### Programs Being Implemented

A few types of tobacco prevention and control programs are being implemented at the National levels, relevant to youth. Began by the Russian Public Health Association (ROZA), public service announcements on the risks of cigarette smoke is now being aired by national, regional, and local television channels [2]. Also, the National Center for Preventive Medicine of the Russian Ministry of Health and the National Cancer Research Center developed a school curriculum for universal smoking prevention among children and adolescents over the last couple of years as well, and 53% of high school youth (in Moscow) report exposure to some type of prevention programming. On the other hand, approximately 40% never discuss smoking in class. A total of 93% of Moscow high school youth do believe that smoking is dangerous (only 65% think smoking "definitely" harms health) and 85% believe that secondhand smoke is dangerous [16].

Various local projects have been implemented. For example, in October of 2005, a noncommercial organization in Novosibirsk won "The Youth Project" competition of socially significant projects of public associations, and received a municipal grant which focuses on the problem of dependence on tobacco, alcohol and drugs among youth, leading to a variety of consequences education efforts among the high schools of Novosibirsk (e.g., showing a video of healthy and smokers' lungs). Similar projects have been conducted in Moscow high schools (e.g., "the Student, Check up the Lungs!") These types of programs are not effective, in general [19]. One promising note is that over 1,700 Moscow-based health professionals have participated on education in smoking cessation for their patients (2003–2006), and over 800 guidelines on smoking cessation for physicians has been distributed. The impact on smoking cessation is not yet known, but this has been a promising direction (including helping physicians, themselves, quit smoking; http://www.ceche.org/programs/pvonis/pvo-nis.htm; [2]; also see http://www.who.dk/tobaccofree/Projects/national/20040603_7).

In a series of studies, Schnoll and colleagues (2006a and 2006b) examined the prevalence of tobacco use, and knowledge of Russian oncologists about smoking cessation, involving 399 Moscow-area cancer patients and 63 oncologists (from a large cancer health center in Moscow). A total of 42% of the cancer patients were smokers, primarily adult male. They exhibited low levels of knowledge about the negative effects of smoking, generally did not intend to quit smoking and exhibited high fatalistic beliefs. The physicians lacked training in smoking interventions, and rarely offered cessation treatment; though they did desire education and training in cessation counseling. Thus, there is a long way to go to establish effective physician-assisted smoking cessation counseling in Russia. Better physician practices as role models and advisors will help result in fewer smokers in subsequent generations.

There also exists the International Quit and Win Contest, World No Tobacco Day (May 31), and International No Smoking Day ("International Day of Refusal of Smoking"; 3^rd ^Thursday of November), which involve media outputs and materials distribution [16] though out Russia. Yearly government expenditures on anti-smoking campaigns organized by the Ministry of Health and Medicine and regional counterparts total approximately $20 million U.S.D. whereas the tobacco industry spends $2 billion U.S.D. per year [16]. Anti-tobacco campaigns for youth often have been industry generated and tend to encourage a forbidden fruit perspective. By suggesting smoking as not appropriate for youth (e.g., BAT's "Smoking? There is no time for this" street advertising campaign, its Retail Access Prevention, Nonsmoking Class Contest, and "Your Choice" prevention education), but is a responsible choice for adults, the tobacco industry is replicating its earlier efforts in the U.S., providing ineffective programming that might even peak curiosity in trying to smoke [4,5,16,19]. Interestingly, a search with "smoking" as a keyword on the Moscow Times web page (http://www.moscowtimes.com; 10-13-06) reveals only one smoking prevention program link http://www.keepkidsfromsmoking.com that is provided by the Lorillard Tobacco Company [19]. In addition, a "This is smoke" (shopzilla.com) link on that same search when clicked leads to several items that can be purchased including cigars. The Russian Public Health Association is one of the only publicly visible organizations that will not collaborate with the tobacco industry [4].

## Results of Focus Groups in Moscow

FINAL REPORT ON THE RITCCPHA-RPHA PROJECT

COMPONENT 1

RESEARCH ON DISCOVERING THE SOCIO-CULTURAL NORMS, BELIEFS AND VALUES OF YOUNG PEOPLE THAT INFLUENCE THEIR DECISION IN WHETHER OR NOT TO SMOKE

The research was carried out in Moscow and two other large regional centres of Russian Federation – Nizhny Novgorod and Petrozavodsk. Data collection was implemented in Moscow by I.A. Demina, M.D., Physician, Ph.D., teacher of Education-pedagogical Complex "Mitino-18", chair of Moscow city chapter of Russian Public Health Association, in Nizhny Novgorod by R.A. Matkivskiy M.D., Physician, Ph.D. Senior researcher of Nizhegorodskiy Research Institute of Children Gastroenterology under Ministry of Health of Russian Federation, and in Petrozavodsk, Karelian Republic by E.N. Vorobyeva M.D., Physician, head of Department of Psychotherapy of Center for Help for Children and Women in city Petrozavodsk, Executive director of Karelian Republic chapter of Russian Public Health Association.

In Moscow there was already a history of collaboration between RPHA and school authorities in conducting the GYTS. This facilitated obtaining permission to conduct the study in Moscow.

The Research Group of Children's Hygiene, Moscow Medical Academy, and others who work with children in Moscow Oblast have expressed interest in the issue of tobacco and youth and have participated in activities in Moscow related to qualitative research.

Representatives from Nizhny Novgorod and Petrozavodsk regional chapters have already attended RPHA workshops on qualitative research and demonstrated an interest in this approach.

### 5.4. Consent

Informed consent was obtained from children, their parents, and schools as required. Confidentiality and anonymity was assured for those participating in the survey and children were assured that they could refuse to participate or withdraw from the study at any time without penalty.

### 5.5. Social, political, gender, or ethical considerations

Before undertaking this project it was very important to have the interest and approval of relevant authorities since having their approval was not just a logistical consideration in Russia.

As was illustrated when conducting the GYTS in that country, their approval increased the likelihood of school official's endorsing participation in the research.

Russia is a country where until recently there were few individual rights and decision making was a very top down process. The idea of involving people, especially youth, in defining social problems and how they should be addressed is a relatively new concept – as is the idea of asking children whether or not they wish to participate in a study.

An emphasis on ethical research, and particularly the ethics of working with children, was an important part of the training workshops. Obtaining truly voluntary consent from youth was an important consideration.

As mentioned earlier, unlike in most Western countries smoking among Russian girls lags behind smoking among young males; but it is nonetheless increasing. This research played an important role in discovering the meaning of tobacco in young girls' lives and insights into why they choose whether or not to smoke and whether a cessation intervention would be suitable for the needs of Russian women.

This information provided a better understanding of how anti-smoking campaigns, or cessation strategy should be developed if they are to be effective in preventing and stopping tobacco use among women in different age groups.

### 5.6. Data Analysis

This study used triangulation and clarification of researcher bias as the methods of verifying the study. Typically the triangulation process involves corroborating evidence from different sources to shed light on a theme or perspective. Researcher bias could be judged a possibility because of researchers' views on the physical dangers of smoking and their experience as health workers, educators, and/or parents. Researchers were requested to reflect on this possibility and check for potential bias on an ongoing basis.

An early and ongoing analysis of the data was carried out to ensure that the study focused on the main issues that are important to participants and is not slanted to researcher bias. Among the activities included in the data analysis process was a discussion of the proposed research to ensure that all those participating in the study clearly understood its purpose and parameters. After each focus group its results were examined. Researchers understood that this is actually part of the analysis process which built upon knowledge that was being accumulated regarding tobacco use among youth. Recordings of interviews and focus groups were also reviewed promptly after the events.

Transcripts were read for general impressions regarding opinions and attitudes, next for specific information.

The Russian research team has made significant progress in collecting the qualitative data for their study of adolescent smoking. The work also included full transcribing of interviews (over 300 pages), analyzing the data, and preparing reports of the findings.

### 5.7. Summary of research findings

• Prior to the survey children were never asked to express their feelings and opinions on tobacco issues.

• Prevalence of smoking is very high. When asked "Who is smoking in your class?" children responded, that it is easier to point to a non-smoker.

• Knowledge on risks of tobacco use among children is poor. There is a lot of myths, for example: "One develops cancer of the lip if does not inhale smoke."

• Social dependence on tobacco and control of negative emotions (school and home stress) are the dominating reasons for tobacco use among children, compared to pleasure, empowering, fullfledged dependence.

• All smoking children conceal their smoking from parents.

• All smoking children attempted quit smoking, but absolute majority failed.

• Smoking accompanies alcohol intake during leisure time spent with peers.

• Some children relieve school stress by smoking during breaks.

• According to children, smoking is not compatible with sports.

• Children believe that smoking compromises memory, intellect and school performance.

• Students hold a view that adults can influence smoking among younger children. Among high school students peer influence is the key.

• There is an opinion that a girlfriend or a boyfriend may be a smoker, but not future husband or wife, because in the latter case the health of the future child will be compromised.

• Children believe that teachers are more tolerant towards smoking among high school students, and are concerned more with fire safety. Many teachers smoke themselves and at the same time attempt to control tobacco use among schoolchildren.

Qualitative data was collected by Andre Demin in three Russion cities in 2003 among children 8–16 year old. These data were compared to focus group data collected by Sussman and others in two U.S. cities in 1997 and 2000 [20]. Both studies addressed similar questions about reasons for beginning to smoke cigarettes, continuing to smoke, reasons for quitting, and about quit attempts, permitting a unique comparison across the two countries. A comparison of these qualitative datasets is summarized as follows.

First trial of cigarettes often occurs away from adults in both countries. However, youth tend to try cigarettes with older friends in Russia but same-age friends in the U.S. Reasons to try tobacco include peer pressure, curiosity, perceived high prevalence of smoking, media influence, and to calm down, common reasons in both countries. Reasons for continued use include cravings, peer pressure, perceptions of tight knit groups that smoke, and to cope with a bad or boring life. These reasons are common in both countries. In addition "to not appear a fool" seems to be a reason suggested in Russia only.

Reasons for quitting include deterioration of physical performance and health, to please parents and other family, non-smoker peer pressure, if restrained from smoking, and to save money in both countries. Quitting due a belief that mental health worsens (become more corrupt and easily upset if a smoker) and to restore moral balance was a response observed in Russia only. Quitting to please a girlfriend or boyfriend or if someone close to the smoker died was observed in the U.S. only. Quit techniques reported being used include throwing out cigarettes, making new friends, use of substitutes (e.g., eating seeds to keep hands and mouth busy), receiving formal treatment, and being really motivated to quit, common to both countries.

Reasons for relapse include dependence, social influence of friends, passive smoke, access to cigarettes, life stresses, and dulling judgment through alcohol use; reasons common to both countries. However, reports of lacking of enough support from family, or of having a moral connection to smoking, was a response observed in Russia only. It is this appeal to a multi-dimensional perspective on "morality", the notion "forbidden" conversations, and of a "Russian idea" perhaps attempting to project a mirror reverse image to the West, that the Russia Federation "swims" around a paradoxical past and present, limiting a focus on eradication of tobacco use to one that protects nonsmokers and smokers and hence perpetuates poor health [11].

## Our Speculations

The authors revisited the current published literature, the focus group data in Moscow, and personal experiences at conferences in Russia and the U.S. We noted that reasons for continued, regular teen smoking that are similar around the world, as was indicated in the focus group section of this paper. Teenagers are rapidly changing in their development, including their brain development, and are curious to take on adult-like roles and experience new activities. Thus, they may tend to smoke and quickly become addicted.

However, there also are factors that may be unique to Russian society. Anecdotal literature suggests that smoking is entrenched historically in Russian Society, as automatic and accepted behavior (e.g., Peter the Great was a heavy smoker; [16]). At the same time, Russian society is in a state of flux without precise social normative reference points in a post-communist era [22]. In fact, various sources argue that smokers' rights are considered equal to nonsmokers' rights in Russia. The emphasis is on individual freedom in personal life habits, with little regard to effects on the overall Russian economy or medical system (which is supposed to be free to all Russians but which is heavily used by smokers relative to non-smokers; e.g., [17]). Thus, there is a tradition of smoking within the context of few social constraints. It would not be surprising if teens smoke within whole families that smoke and share cigarettes, knowing that regulations against them smoking exist but not really being concerned. In addition, while being exposed to a demanding elementary and high school education (literacy in Russia is 98%), opportunities for engrossing jobs later may be perceived as limited. Youth may smoke, not anticipating great value of a long life.

Trying to motivate teens to quit smoking may be rather difficult. The tobacco industry dominates tobacco prevention efforts, creating a forbidden fruit perspective [19]. Among adults, there may be relatively fewer quit attempts in Russia than in other countries (e.g., 40% versus 60% in Finland; see [9]). That is, adult role models of smoking cessation, even among health professionals, may be lacking. Further compounding these social learning factors, some public reports depict Russians as lacking in hope, that all efforts are in vain, that there is no economically meaningful future for them, and that the fates will decide the duration of one's life [12,22,17]. If this indeed is a widespread attitude (the Russian Federation suicide rate is second highest in the world; [12]), and if youth adopt these attitudes, it would not be surprising if relatively few youth made serious attempts to quit smoking. Indeed, one may conjecture that fatalism may limit one's commitment to change one's behavior because individual behaviors would not be perceived as affecting the course of life [22].

One may speculate that perhaps since females in Russia smoke much less than males they may provide clues as to how to decrease prevalence of smoking and increase prevalence of quitting among teens. Among females, smoking has been considered as an activity that is not appropriate to take on. People who smoke may be frowned on by others, and may feel demoralized and disfigured. Husbands often forbid their wives to smoke. (This can create a paradox, of course. Women may perceive smoking as a means to be free of male oppression, as well as a means to appear mysterious, independent, and sensual.) In addition, women may be relatively well-aware, or less fatalistic, regarding health consequences of smoking.

## Conclusion

The Russian Federation must contend with widespread tobacco use among males and an apparent increase in tobacco use among young females. There are many reasons that Russians take up smoking, continue to smoke, and fail to quit. [4] and [16] have suggested many ways to decrease smoking prevalence and increase quitting. Certainly, ratification of the Framework Convention on Tobacco Control is one of these means. In May of 2003, the member countries of the World Health Organization adopted the FCTC [6]. The FCTC intends to be an international agreement that would commit countries to adopt strong tobacco control policies. It entered into force on Februrary 27, 2005. A total of 168 countries have signed the treaty, and currently 115 have become Parties in the treaty. Signing the treaty in itself does not legally bind the countries to implement the provisions of the FCTC. In order for them to be legally bound the countries must become Parties to the treaty. Most countries become Parties to the treaty by ratifying it. Ultimately, therefore, the responsibility is that of the national governments to implement the FCTC and protocols. How effective the FCTC will be in reversing the tobacco epidemic will be determined by how fully governments implement the obligations contained in the FCTC.

Key provisions in the treaty encourage countries to (a) enact comprehensive bans on tobacco advertising, promotion and sponsorship; (b) obligate the placement of rotating health warnings on tobacco packaging that cover at least 30 percent (but ideally 50 percent or more) of the principal display areas and can include pictures or pictograms; (c) ban the use of misleading and deceptive terms such as "light" and "mild"; (d) protect citizens from exposure to tobacco smoke in workplaces, public transport and indoor public places; (e) combat smuggling, including the placing of final destination markings on packs; and (f) increase tobacco taxes (see Table 1). The FCTC also contains other measures designed to promote and protect public health, such as mandating the disclosure of ingredients in tobacco products, providing treatment for tobacco addiction, encouraging legal action against the tobacco industry, and promoting research and the exchange of information among countries.

Due to a "change" in organizational structure when involved in a political protocol that targeted consideration of the FCTC, the Russian Federation has neither signed nor ratified the treaty [5]. "Smokers' rights have a strong voice in Russia today – and individual freedom and enterprise is not well integrated with community responsibility. It is at that point at which nonsmokers' rights gather momentum, the tobacco industry is widely fought through public protests, and evidence-based programming is developed and implemented that teen tobacco use cessation can become more a reality. Particularly by great rises in price of cigarettes, enforcement of no smoking laws for minors, and implementation of good teen cessation practices great strides will be made.

## Appendix 1

THE RUSSIAN FEDERATION THE FEDERAL LAW ABOUT RESTRICTION OF SMOKING OF TOBACCO

It is accepted by the State Duma On June, 21st, 2001

It is approved by Council of Federation On June, 29th, 2001

The present Federal law defines legal bases of restriction of smoking of tobacco with a view of decrease in desease of the population.

Regulation of activity on manufacture of tobacco products, wholesale trade in tobacco products and retail of tobacco products

1. Activity on manufacture of tobacco products, wholesale trade in tobacco products is a subject to licensing according to the legislation of the Russian Federation.

2. Manufacture, import, wholesale trade and retail of the cigarettes mismatching hygienic norms of the maintenance in a smoke of nicotine and pitch, the approved authorized federal enforcement authority in the field of public health services are forbidden. Thus parameters of the maintenance of harmful substances cannot exceed:

For cigarettes with the filter – the maintenance in a smoke of a cigarette of pitch 14 milligram on a cigarette and nicotine 1,2 milligrams on a cigarette; For cigarettes without the filter – the maintenance in a smoke of a cigarette of pitch 16 milligram on a cigarette and nicotine 1,3 milligrams on a cigarette.

3. Each packing (pack) of tobacco products should contain precautionary inscriptions about harm of smoking of tobacco – the basic precautionary inscription about harm of smoking of tobacco, an additional inscription about harm of smoking of tobacco and an information inscription about the maintenance of pitch and nicotine in a smoke of a cigarette.

On one greater party of packing (pack) of tobacco products the basic precautionary inscription about harm of smoking of the tobacco, approved by federal enforcement authority on public health services should be placed. On other greater party of packing (pack) of tobacco products one additional inscription about harm of smoking of tobacco in conformity with following rules should be placed:

Each manufacturer of tobacco products chooses from the list approved by federal enforcement authority on public health services, four variants of additional inscriptions about harm of smoking of tobacco;

Each of the chosen variants of additional inscriptions about harm of smoking of tobacco should be placed on equal quantity of packings (packs) of tobacco products.

The basic precautionary inscription about harm of smoking of tobacco and an additional inscription about harm of smoking of tobacco (without taking into account the instruction of the author of the prevention on harm of smoking – federal enforcement authority on public health services) on packing (pack) of tobacco products should borrow not less than four percent of the area of each greater party of packing (pack) of tobacco products.

On one of lateral faces of each packing (pack) of cigarettes the information inscription about the maintenance of pitch and nicotine in a smoke of a cigarette according to state standards also should be placed. The specified inscription should borrow not less than four percent of the area of a lateral face of packing (pack) of cigarettes.

Following demands are made to inscriptions on packing (pack) of tobacco products:

The inscription should be precise and easily readable;

The inscription should be located so that to provide integrity of an inscription at opening packing (pack) of tobacco products;

The inscription should not be printed on a transparent wrapping film or on any other external packing material.

4. Piece retail of cigarettes and cigarettes, and also sale of tobacco products with use of automatic devices are forbidden retail of cigarettes with the maintenance less than 20 pieces of cigarettes in each packing (pack).

5. Retail of tobacco products in the organizations of public health services, the educational organizations and the organizations of culture, and also in the +,-./01'/%)"-sports organizations Is forbidden.

Clause 4. Prohibition of retail of tobacco products to the persons who have not reached age of 18 years

1. In territory of the Russian Federation retail of tobacco products to the persons who have not reached age of 18 years is not supposed.

2. Infringement of position of item 1 of present clause entails attraction to the administrative responsibility according to the legislation.

Clause 5. Advertising of tobacco and tobacco products

Advertising of tobacco and tobacco products is carried out according to the legislation of the Russian Federation on advertising.

Clause 6. Prohibition of smoking of tobacco on workplaces,

In city, suburban transport and on air transport,

In the closed sports constructions, the organizations of public health services,

The educational organizations and the organizations of culture, the premises borrowed by bodies of the government

1. With a view of decrease in harmful influence of a tobacco smoke smoking tobacco on workplaces, in city and suburban transport, on air transport is forbidden at duration of flight less than three hours, in the closed sports constructions, the organizations of public health services, the educational organizations and the organizations of culture, the premises borrowed by bodies of the government, except for smoking tobacco in specially allocated smoking areas of tobacco.

2. The duty is assigned to the employer on equipment of specially allocated smoking areas of tobacco.

3. Infringement of positions of given clause entails attraction to the administrative responsibility according to the legislation.

Clause 7. Propagation of knowledge of harm of smoking of tobacco

1. Federal enforcement authorities on public health services, federal enforcement authorities by formation and federal enforcement authorities on culture are obliged to carry out on a regular basis through mass media propagation of knowledge of harm of smoking of tobacco.

2. With a view of realization of positions of the present Federal law general educational programs and professional educational programs should contain the sections, concerning studying of influence on an organism of the person of smoking of tobacco. Are not supposed demonstration of smoking of tobacco in again created television films, in films and performances if such action is not an integral part of an art plan, and demonstration of smoking of tobacco public and politicians in mass media.

Clause 8. Measures on restriction of smoking of tobacco

The government of the Russian Federation develops measures on restriction of smoking of tobacco and provides their realization.

Clause 9. Reduction of normative legal certificates conformity with the present Federal law

To the president of the Russian Federation and the Government of the Russian Federation to bring the normative legal certificates into accord with the present Federal law.

Clause 10. Coming into force of the present Federal law

The present Federal law inures in six months from the date of its official publication, except for items 2 and 3 clauses 3 and item 2 of clause 7 of the present Federal law.

Item 2 of clause 3 of the present Federal law regarding an interdiction of manufacture and import of tobacco products inures since January, 1st, 2003. Item 2 of clause 3 of the present Federal law regarding an interdiction of wholesale trade and retail of tobacco products inures since January, 1st, 2004. Item 3 of clause 3 of the present Federal law inures since January, 1st, 2003. Item 2 of clause 7 of the present Federal law inures in one year from the date of official publication of the present Federal law.

The president

The Russian Federation

V. 23456

## Competing interests

The authors declare that they have no competing interests.
